# HIV, multidrug-resistant TB and depressive symptoms: when three conditions collide

**DOI:** 10.3402/gha.v7.24912

**Published:** 2014-09-09

**Authors:** Mrinalini Das, Petros Isaakidis, Rafael Van den Bergh, Ajay MV Kumar, Sharath Burugina Nagaraja, Asmaa Valikayath, Santosh Jha, Bindoo Jadhav, Joanna Ladomirska

**Affiliations:** 1 Médecins Sans Frontières (MSF) OCB, India; 2 Médecins Sans Frontières (MSF) OCB, Brussels, Belgium; 3 International Union Against Tuberculosis and Lung Disease (The Union), South-East Asian Regional Office, New Delhi, India; 4 ESIC Medical College and PGIMSR, Bangalore, India; 5 K. J. Somaiya Medical College Hospital And Research Centre, Mumbai, India

**Keywords:** MDR-TB, HIV, depressive symptoms, depression, PHQ-9, operational research, counselling, psychiatric illnesses, India

## Abstract

**Background:**

Management of multidrug-resistant TB (MDR-TB) patients co-infected with human immunodeficiency virus (HIV) is highly challenging. Such patients are subject to long and potentially toxic treatments and may develop a number of different psychiatric illnesses such as anxiety and depressive disorders. A mental health assessment before MDR-TB treatment initiation may assist in early diagnosis and better management of psychiatric illnesses in patients already having two stigmatising and debilitating diseases.

**Objective:**

To address limited evidence on the baseline psychiatric conditions of HIV-infected MDR-TB patients, we aimed to document the levels of depressive symptoms at baseline, and any alteration following individualized clinical and psychological support during MDR-TB therapy, using the Patient Health Questionnaire-9 (PHQ-9) tool, among HIV-infected patients.

**Design:**

This was a retrospective review of the medical records of an adult (aged >15 years) HIV/MDR-TB cohort registered for care during the period of August 2012 through to March 2014.

**Results:**

A total of 45 HIV/MDR-TB patients underwent baseline assessment using the PHQ-9 tool, and seven (16%) were found to have depressive symptoms. Of these, four patients had moderate to severe depressive symptoms. Individualized psychological and clinical support was administered to these patients. Reassessments were carried out for all patients after 3 months of follow-up, except one, who died during the period. Among these 44 patients, three with baseline depressive symptoms still had depressive symptoms. However, improvements were observed in all but one after 3 months of follow-up.

**Conclusion:**

Psychiatric illnesses, including depressive symptoms, during MDR-TB treatment demand attention. Routine administration of baseline mental health assessments by trained staff has the potential to assist in determining appropriate measures for the management of depressive symptoms during MDR-TB treatment, and help in improving overall treatment outcomes. We recommend regular monitoring of mental health status by trained counsellors or clinical staff, using simple, validated and cost-effective tools.

The human immunodeficiency virus (HIV) is a driving force behind the global burden of tuberculosis (TB) and the development of drug-resistant tuberculosis (DR-TB). Management of multidrug-resistant TB (MDR-TB) patients co-infected with HIV is highly challenging. With growing evidence showing psychiatric illnesses such as depression, anxiety and psychosis to be associated with MDR-TB (1, 2) and HIV (3), mental health care for patients with these two stigmatising and debilitating diseases demands attention.

An MDR-TB patient co-infected with HIV is subject to long and potentially toxic treatment that may make the patient debilitated (4), stressed, and de-motivated. Additionally, dependence on the family for support, discrimination, and/or financial problems can further influence the mental wellbeing of patients. These may develop into a number of psychiatric illnesses such as anxiety and depressive disorders, which may lead to suboptimal adherence to both antiretroviral (3, 5) and anti-TB treatments (2), and ultimately poor treatment outcomes.

The baseline mental health characteristics of patients may determine the development of subsequent psychiatric conditions. A mental health assessment before MDR-TB treatment initiation may thus assist in better management of psychiatric illnesses and improve treatment outcomes (6, 7). While psychiatric adverse events occurring during the course of treatment have been well documented (1), limited evidence exists on the baseline psychiatric conditions of HIV-infected MDR-TB patients. To address this evidence gap, we aimed to document the levels of depressive symptoms at baseline, and any alteration following clinical and psychological support during MDR-TB treatment, among a cohort of HIV-infected MDR-TB patients attending a clinic in Mumbai, India.

## Methods

### Study design

This study involves a retrospective review of the medical records of an adult cohort (aged >15 years) of MDR-TB patients co-infected with HIV.

### Setting and study population

The Médecins Sans Frontières (MSF) Clinic in Mumbai, India provides individualized care to MDR-TB patients, as described elsewhere (8). Psychological assessments are carried out using the Patient Health Questionnaire-9 (PHQ-9) tool. The PHQ-9 tool, a nine-item checklist, has been widely used for the assessment of depressive symptoms (9), including in TB patients (10). Together with other clinical assessments, it is administered by trained MSF counsellors at baseline (i.e. before or on the day of MDR-TB treatment initiation) and every 3 months during follow-up after MDR-TB treatment initiation. For the purpose of our study, any patient found to have a score >4 using this tool was defined as having depressive symptoms. The levels of depressive symptoms were classified as mild (PHQ-9 score: 5–9), moderate (10–14) , moderately severe (15–19) and severe (20–27) (10). A patient with a PHQ-9 score <5 was considered to be ‘without depressive symptoms’.

All the patients are managed by a multidisciplinary team consisting of trained physicians, nurses, social workers and psychologists (1). Treatment education is provided to every patient, along with their family member/partner, during treatment initiation and follow-up. Treatment is provided to patients considering pharmacological interactions between medications of three illnesses: alteration of doses of medications and/or MDR-TB regimen itself, if required. Individual counselling sessions are carried out for patients with depressive symptoms, addressing stress, poor adherence, and/or low motivation. A psychiatrist is on-board for advanced clinical care of these patients. If needed, patients receive psychiatric consultations with/without medications during the treatment.

All HIV-infected MDR-TB patients aged >15 years registered at the MSF clinic for treatment from August 2012 to March 2014 were included in the study. Patients without depressive symptoms assessment before MDR-TB treatment initiation using PHQ-9 tool, were excluded from the analysis.

### Data collection and analysis

Demographics, social and clinical data including age, sex, literacy level, marital status, family income, family support, previous TB episodes, TB resistance pattern and antiretroviral treatment were collected from routine programme data and from there, data were double-entered using EpiData (version 3.1, EpiData Association, Odense, Denmark) and validated. Baseline and 3-month follow-up data of ‘depressive symptoms’ for each patient were analysed.

### Ethics

The study received approval from the MSF Ethics Review Board in Geneva, Switzerland and the Ethics Advisory Group of the International Union Against Tuberculosis and Lung Disease, Paris, France. As this was a study of routinely collected monitoring data, informed consent from the patients was not obtained. The named ethics committee waived the need for consent.

## Results

**Fig. 1:**
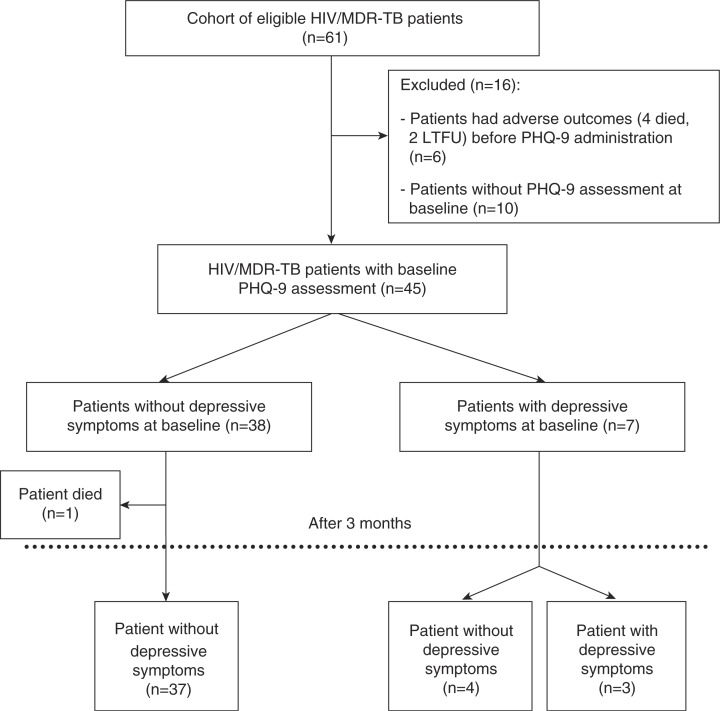
HIV/MDR-TB patients with depressive symptoms, using the PHQ-9 tool in an MSF clinic in Mumbai, India (August 2012–March 2014). HIV/MDR-TB: HIV and multi-drug resistance tuberculosis co-infection; LTFU: loss-to-follow-up; PHQ-9: Patient Health Questionnaire-9

During the study period, a total of 61 HIV/MDR-TB patients were registered for care in clinic (Fig. 1). Of these, 45 patients underwent baseline depressive symptoms assessment using the PHQ-9 tool: seven (16%) patients had depressive symptoms while 38 did not have baseline depressive symptoms. Baseline depressive symptom levels could not be assessed for the other 16 patients due to either the severity of their illness at treatment initiation (6), or the low priority accorded to the assessment by the counsellors (10).

Out of the seven patients with baseline depressive symptoms, five had previous TB episodes (Table 1). Two patients had MDR-TB, three had pre-extensively drug-resistant TB (Pre-XDR-TB) and one patient each had extensively drug-resistant TB (XDR-TB) and extremely drug-resistant TB (X-XDR-TB), respectively. Individualized psychological and clinical support was offered/administered to patients (see Table 1).

**Table 1:** HIV/MDR-TB patients with baseline depressive symptoms attending MSF clinic, Mumbai, India (August 2012–March 2014)

Case	Age	Sex	Family support	Number of previous TB episode(s)	Patient on ART during treatment	TB Resistance pattern	Baseline depressive symptoms status (PHQ-9 score out of 27)	Support administered	Intermediate mental health outcomes (after 3 months) (PHQ-9 score out of 27)
1	47	F	Present	3	Yes	MDR	Mild (5)	- Individual counselling (motivation & emotional)	Improved (0)
2	35	M	Absent	2	Yes	Pre-XDR	Severe (20)	- Psychiatric consultation with medication - Individual counselling (motivation & emotional)	Same as before (18)
3	47	M	Present	1	Yes	XDR	Mild (6)	- Individual counselling session (motivation) - Treatment education involving AE	Improved (0)
4	36	M	Present	2	Yes	X-XDR	Moderately severe (16)	- Psychiatric consultation with medication - Individual counselling session (motivation)	Improved (12)
5	27	F	Absent	0	Yes	Pre-XDR	Moderate (11)	- Psychiatric consultation without medication - Emotional support	Improved (3)
6	35	M	Present	1	Yes	Pre-XDR	Moderate (11)	- Individual counselling session (motivation)	Improved (9)
7	16	F	Present	0	Yes	MDR	Mild (5)	- Emotional support	Improved (4)

M: male; F: female; TB: tuberculosis; AE: adverse events during treatment; MDR-TB: multidrug-resistant tuberculosis; Pre-XDR-TB: pre-extensively drug-resistant tuberculosis; defined as MDR-TB case whose recovered *M. tuberculosis* isolate is resistant to at least isoniazid, rifampicin, and either a fluoroquinolone or a second-line injectable anti-TB drug; XDR-TB: extensively drug-resistant tuberculosis; X-XDR-TB: Extremely drug-resistant tuberculosis; defined as MDR-TB case whose recovered M. tuberculosis isolate is resistant to all first-line and second-line anti-TB drugs.

Reassessments were carried out using the PHQ-9 tool for all patients after 3 months of follow-up (Fig. 1), except one, who died during the period. Among these 44 patients, three patients (with baseline depressive symptoms) still had depressive symptoms. However, improvements in depressive-symptoms were observed in all but one patient (Table 1).

## Discussion

Our study showed that about one-fifth of our HIV/MDR-TB co-infected patients had baseline depressive symptoms, which were managed with individualized psychological and clinical support interventions.

Psychiatric illnesses have previously been documented as an adverse event during MDR-TB treatment (1), and have been associated with poor treatment outcomes in TB patients (7, 11). Depression is one of the most common psychiatric illnesses found in both HIV-infected patients (3) and those under MDR-TB treatment (2). However, despite being described as a major psychiatric condition in pre-therapy MDR-TB patients (2), no baseline assessments of depression have been reported among HIV/MDR-TB patients.

In our study, all but one patient with baseline depressive symptoms improved after 3 months of MDR-TB treatment. Further, none of the patients had newly developed depressive symptoms after 3 months of follow-up. Thus, routine administration of baseline and follow-up mental health assessments in MDR-TB patients has the potential to assist in identifying those with psychiatric illness and in need of appropriate measures for their management during MDR-TB treatment, which could in turn improve overall treatment outcomes (6, 7).

The treatment of depressive symptoms in HIV/MDR-TB patients needs strategic selection of medications and/or dosages for all three illnesses, including consideration of possible pharmacological interactions. Some anti-depressants, when administered to HIV-infected patients, may exacerbate certain symptoms associated with HIV (12). Similar pharmacological interactions have been documented between isoniazid and anti-depressants (13) for tuberculosis patients. Thus, there is an urgent need for simple, low pill burden, patient friendly regimens for HIV/MDR-TB co-infected patients suffering from depressive symptoms.

A strength of this study was the use of a standardized, validated tool (PHQ-9) (9) by trained MSF counsellors for the assessment of depressive symptoms. While the small sample size is a limitation, the patients represent part of the largest HIV/MDR-TB cohort in India. Another limitation was that baseline depressive symptom assessments were not carried out for all patients, due to severity of the patients’ condition at registration in some or lack of priority accorded by the counsellors in other cases. The team felt that more attention was required to first hospitalize/stabilize these patients (clinically or/and emotionally), than carrying out their psychological assessment using the tool. The latter highlights a major challenge towards administration of a PHQ-9 tool, and the need for well-trained counsellors in a programmatic setting; the former suggests that, as patients in a very severe condition may be more likely to suffer from depressive symptoms, the prevalence of depressive symptoms may be underreported in our study.

Regular monitoring of the mental health status at baseline and during MDR-TB treatment may help treatment providers, patients and family members in appropriate management of a person's condition during the different stages of illness. Simple, cost-effective, and easy-to-administer tools are required in order to facilitate accurate monitoring of these patients. A number of mental health assessment tools (6, 7, 11) including the PHQ-9 checklist (10, 14) have been identified for tuberculosis patients. However, concerns have been raised about the appropriateness of PHQ-9 checklist as a diagnostic tool (15) and about its use for the Asian Indian population (16). A setting-specific, validated tool is a key requirement for accurate mental health assessments in MDR-TB patients.

Psychiatric co-morbidities are not a contra-indication to MDR-TB treatment, as described by Vega et al. (2). However, caution should be exercised during the treatment of depressive symptoms in MDR-TB patients receiving a regimen inclusive of cycloserine: it may be difficult to differentiate between drug-induced psychological complaints (2) and/or identification of pharmacological interactions between MDR-TB medications and anti-depressants. Care should be individually tailored to help patients cope with the combined burden of depressive symptoms in addition to their existing debilitating, stigmatising diseases of HIV and MDR-TB (4).

## Conclusion

Psychiatric illnesses during MDR-TB treatment, including depressive symptoms, demand attention. The clinical staff involved in management of MDR-TB patients should be trained to administer appropriate mental health assessment tools, especially at baseline, so that psychiatric disorders can be identified early. We recommend regular monitoring of mental health status by trained counsellors or clinical staff, using simple, validated and cost-effective tools.
